# Influence of Nanoparticle Content and Cross-Linking Degree on Functional Attributes of Calcium Alginate-ZnO Nanocomposite Wound Dressings

**DOI:** 10.3390/membranes15040108

**Published:** 2025-04-01

**Authors:** Sergio Henrique Toledo e Silva, Andrea Cristiane Krause Bierhalz, Ângela Maria Moraes

**Affiliations:** 1 School of Chemical Engineering, University of Campinas, Avenida Albert Einstein, 500, Cidade Universitária Zeferino Vaz, Campinas 13083-852, São Paulo, Brazil; sergio.toledo@ivv.fraunhofer.de; 2 Department of Textile Engineering, Federal University of Santa Catarina, Rua João Pessoa 2750, Blumenau 89036-004, Santa Catarina, Brazil

**Keywords:** ZnO nanoparticles, alginate membranes, antimicrobial activity, mechanical properties

## Abstract

Alginate-ZnO nanoparticles (ZnO_nano_) composite wound dressing membranes were prepared with two different ZnO_nano_ concentrations (0.03 and 0.20 g ZnO/g sodium alginate) and cross-linked with two different calcium treatments (low and high Ca^++^concentration) to evaluate the influence of nanoparticle content and cross-linking degree on membrane attributes. ZnO_nano_ addition did not significantly alter the mechanical properties, water vapor permeability, swelling degree in water and the alginate amorphous nature of the nanocomposite membranes. The increase in cross-linking degree, on the other hand, altered the microstructure of the membranes, increased the tensile strength and reduced the water vapor permeability of the nanocomposite membranes. The presence of ZnO_nano_ in alginate membranes granted them antibacterial activity in vitro against *Pseudomonas aeruginosa* and *Staphylococcus aureus* and substantially increased the absorption capacity in phosphate buffer and fetal bovine serum solutions, validating their potential use as wound dressings.

## 1. Introduction

Polysaccharides are attractive options of raw materials for developing biomaterials due to their biocompatible, non-toxic and biodegradable properties and also to their abundant availability as natural resources. Among these natural polymers, alginate has been given considerable attention due to its ability to form strong hydrogels in the presence of divalent ions, such as calcium.

Alginate can be described as a block copolymer composed by β-d-manuronic acid (M residues) and α-l-guluronic acid (G residues) [1]. The gelation mechanism of alginate chains in the presence of calcium can be described by the egg box model, in which the calcium ions are accommodated in cavities formed by two parallel helicoidal guluronate sequences [2,3]. Due to their cross-linking properties, alginates are versatile macromolecules for the manufacture of a range of materials such as particles, fibers, hydrogels, thin films and membranes, favoring the development of novel materials for biomedical applications [4].

Alginate matrices can be used to incorporate a wide range of ingredients to grant them specific functions, such as antimicrobial, antioxidant or anti-inflammatory properties. These additives can be organic acids, antibiotics, inorganic compounds and nanoparticles [5], e.g., silver and zinc oxide nanoparticles.

Zinc oxide is an inorganic compound with a wide range of applications, as in the production of pigments, cosmetics, pharmaceuticals and chemical sensors. It is one of the five zinc compounds approved by the Food and Drug Administration [6]. In addition, a number of published works has described the activity of ZnO nanoparticles (ZnO_nano_) against both Gram negative and Gram positive bacteria when dispersed in water or when incorporated in polymeric matrices [7,8]. Therefore, the use of ZnO_nano_ is of particular interest in the development of antimicrobial biomaterials, such as wound dressings for infected ulcers, as these lesions often exhibit impaired healing, strain health care systems and diminish patients’ quality of life [9].

The potential of ZnO nanoparticles to improve distinctive qualities of polymeric nanocomposite membranes has already been described in the literature, e.g., for matrices consisting of chitosan [10], chitosan/poly(vinyl alcohol) (PVA) [11] and chitosan/poly(ethylene glycol) (PEG) [12], among other examples. Matrices based on alginate and ZnO nanoparticles have shown promising results in wound healing applications. For instance, a clinical study involving patients with diabetic foot ulcers demonstrated that the incorporation of ZnO nanoparticles into a calcium alginate dressing significantly improved the healing process, promoting epithelialization and healthy tissue granulation [13]. Dodero et al. [14] demonstrated that ZnO nanoparticles incorporated into electrospun alginate membranes crosslinked with Ca^2^⁺, Ba^2^⁺ or Sr^2^⁺ exhibited strong antibacteriostatic and antibacterial properties, while maintaining their cytocompatibility towards L929 murine fibroblast and HaCaT humankeratinocyte cell lines. Wang et al. [15] developed a bi-layered hydrogel based on sodium alginate cross-linked with calcium and showed that the mechanical properties, antibacterial rate and in vivo wound healing rate increases with ZnO nanoparticle content. Nevertheless, the influence of nanoparticle content and the cross-linking degree on alginate membrane properties has not yet been systematically reported, particularly in terms of assessing its suitability for biomedical applications, which constitutes the novelty of this work.

Therefore, this work aimed to develop and characterize alginate and zinc oxide nanocomposite membranes cross-linked with different contents of calcium ions and to evaluate their potential as wound dressings. The nanocomposite membranes were characterized by scanning electron microscopy, X-ray diffraction, Fourier transform infrared spectroscopy and thermogravimetric analysis. The in vitro antimicrobial activity against *Pseudomonas aeruginosa* and *Staphylococcus aureus* was also determined.

## 2. Materials and Methods

### 2.1. Materials

Sodium alginate of medium viscosity (lot # SLBM3663V), with an average molar mass of 90 kDa and high content of M blocks (mass fraction of 0.6), and ZnO_nano_ with average mean diameter below 100 nm were purchased from Sigma-Aldrich (Saint Louis, MO, USA). Calcium chloride dihydrate from Merck Inc. (Darmstadt, Germany) was used as a cross-linking agent, and glycerol (from Synth, Campinas, Brazil) as plasticizer. All reagents were of at least analytical grade and were used as received, without any further purification procedures. *Pseudomonas aeruginosa* (ATCC 27853) and *Staphylococcus aureus* (ATCC 6538) strains used in the in vitro antimicrobial activity were acquired from Fundação André Tosello (Campinas, SP, Brazil).

### 2.2. Preparation of the Nanocomposite Membranes

The alginate-ZnO nanocomposite membranes were prepared by casting [16], with adaptations. At first, 0.18 or 1.2 g of ZnO_nano_ (corresponding to mass ratios of 3 and 20% in reference to total sodium alginate amount) were dispersed in 400 mL of deionized water and vigorously stirred at 24,000 rpm with an Ultra Turrax (IKA, Staufen, Germany) for 5 min. The mass ratio of ZnO nanoparticles to sodium alginate was defined based on the results of the desirability function analysis of Espitia et al. [17]. Afterwards, the mixture was sonicated for 30 min in an ultrasound bath at 200 W (Ultrassonic Cleaner, São Paulo, Brazil). Then, 3.6 g of glycerol was added to the dispersion, followed by the slow addition of 6.0 g of sodium alginate under mechanical stirring (900 rpm) (Tecnal, São Paulo, Brazil).

The mixture was heated up to 70 °C, and 30 mL of a solution of CaCl_2_·2H_2_O at 0.8% (*w*/*v*) were added at the rate of 0.6 mL/min using a peristaltic pump (Masterflex C/L, Studio City, CA, USA). Next, the mixture was cast into 225 cm^2^ poly(methyl methacrylate) plates (50 g/plate) and dried at 40 °C in a convection oven (Tecnal, São Paulo, Brazil). After 24 h, the membranes were detached from the casts and stored in a chamber at 52% of relative humidity. These membranes, produced to achieve a low cross-linking degree, were denominated as L-membranes.

To produce highly cross-linked nanocomposite membranes (referred to as H-membranes), the membranes produced as described before were afterwards immersed for 20 min in 50 mL of 3% (*w*/*v*) calcium chloride dihydrate aqueous solution containing 3% (*w*/*v*) of glycerol and then immersed in glycerol aqueous solution at 3% (*m*/*v*) for 1 min to wash away the excess of calcium. At last, these membranes were dried using an in-house manufactured drying tunnel for 3 h at room temperature (around 25 °C).

Control membranes with low and high cross-linking degrees were prepared similarly, but without the ZnO_nano_ addition and dispersion steps. All membranes were kept at 52% relative humidity for 72 h before characterization. A total of six different formulations were prepared, as listed in Table 1.

### 2.3. Nanocomposite Membranes Characterization

All characterization assays were performed in triplicate, unless otherwise stated.

#### 2.3.1. Nanocomposite Microstructure

The surface and cross-section microstructure of the nanocomposite membranes were evaluated using a scanning electron microscope (SEM, Quanta 650 FEG, FEI, Hillsboro, OR, USA) with an acceleration voltage of 5 kV. The specimens were coated with gold and palladium before SEM observations.

The topography of the surface of the membranes was examined with an atomic force microscope (AFM, Nanosurf Flex, Nanosurf, Liestal, Switzerland), using the intermittent contact mode in random areas of 10 × 10 μm^2^.

#### 2.3.2. X-Ray Diffraction (XRD)

X-ray diffraction patterns were recorded with an X-ray diffractometer (X’Pert PW 3050, Philips Analytical, Drachten, The Netherlands), using a Cu-Kα radiation operated at 40 kV and 40 mA with scanning diffraction angles from 5 to 75° (2θ).

#### 2.3.3. Fourier Transform Infra-Red Spectroscopy (FT-IR)

Nanocomposite and control membranes FT-IR spectra were obtained with a spectrophotometer (Nicolet, Thermo-Scientific, Waltham, MA, USA) operated at resolution of 4 cm^−1^. The spectra were recorded at the wave number range of 650–4000 cm^−1^.

#### 2.3.4. Thermogravimetric Analysis (TGA)

Dynamic TGA was accomplished using a thermogravimetric analyzer (TGA-50M, Shimadzu, Kyoto, Japan) operated from 20 to 600 °C at a heating rate of 10 °C/min under a nitrogen gas purge of 40 mL/min.

#### 2.3.5. Membrane Thickness and Mechanical Properties

The thickness of the membranes was measured with a digital micrometer (MDC-25S; Mitutoyo, Kawasaki, Japan) at 10 random positions.

Mechanical properties were determined following the ASTM D882 method [18]. Tensile strength (TS), elongation at break (E), and Young’s modulus (Y) were determined at 25 °C (±1 °C) using a texturometer (TA.XT2, Stable Microsystems SMD, Surrey, UK), with a cell load of 50 kg, a gauge length of 5 cm and a crosshead speed of 0.1 cm/s. The results are expressed as the average of 10 specimen analyses.

#### 2.3.6. Color Properties and Opacity

Color parameters and opacity (OP) of the membranes were measured with a colorimeter (Colorquest II; Hunterlab, Reston, VA, USA), operating in the transmittance mode with CIELab standards and the Hunterlab method, as described by De Souza et al. [19].

The color parameters ΔE* (total color difference between nanocomposite and control membranes with the same cross-linking treatment), Hue and Chroma, were calculated according to equations Equation (1), Equation (2) and Equation (3), respectively, based on the values determined for L* (lightness), a* (ranging from red to green) and b* (yellow to blue):

(1) $$\Delta E^{*}={\left[{\left(L^{*}-{L_{c}}^{*}\right)}^{2}+{\left(a^{*}-{a_{c}}^{*}\right)}^{2}+{\left(b^{*}-{b_{c}}^{*}\right)}^{2}\right]}^{0.5}$$

(2) $$Hue=tan^{-1}\left(\frac{b^{*}}{a^{*}}\right)$$

(3) $$Chroma={\left[{\left(a^{*}\right)}^{2}+{\left(b^{*}\right)}^{2}\right]}^{0.5}$$

Calculations were made for D-65 illuminant and at 10° observation intervals. The subscript *c* indicates the control membrane parameters, measured similarly as for the nanocomposite membranes. Results are expressed as the average of five replicates.

#### 2.3.7. Water Vapor Permeability and Water Vapor Transmission Rate

Water vapor permeability (WVP) and water vapor transmission rate (WVTR) were determined according to the E95-96 method [20]. The sample to be analyzed was sealed over a circular opening (46.24 cm^2^) of a Plexiglas^®^ permeation cell filled with anhydrous calcium chloride (condition of 0% relative humidity, RH). The cell was placed in a hermetically closed chamber maintained at 75% RH with saturated NaCl solution, and its mass gain was measured periodically during 3 days. The *WVP* and *WVTR* were calculated using Equation (4) and Equation (5), respectively:

(4) $$WVP=\frac{G\cdot \delta }{A\cdot \Delta RH\cdot P_{w}}$$

(5) $$WVTR=\frac{G}{A}$$

where *G* is the permeation rate (g/s) calculated by linear regression of the mass gain versus time, *A* is the permeation area (m^2^), *δ* is the membrane average thickness (m), Δ*RH* is the difference in relative humidity (0.75) and *Pw* is the partial water vapor pressure at the test temperature (3.167 kPa).

#### 2.3.8. Moisture Content

The moisture content of the membrane samples with 2.5 cm diameter was gravimetrically determined in a vacuum oven (Lab-Line, Squaroid, St. Louis, MO, USA) at 105 °C for 24 h.

#### 2.3.9. Swelling in Physiological Solutions

The swelling tests were performed by immerging the membrane samples (dry disc with 2.5 cm diameter, with initial mass previously determined, *m_i_*) in three different fluids: water, phosphate-buffered saline (PBS) and fetal bovine serum (FBS). After 24 h, the control and nanocomposite membranes were withdrawn and gently blotted with absorbing paper to remove the excess of fluid, and their final mass (*m_f_*) was once again measured. The swelling degree was calculated using Equation (6):

(6) $$SD=\left(\frac{m_{f}-m_{i}}{m_{i}}\right)\times 100$$

#### 2.3.10. In Vitro Antimicrobial Activity

In vitro antimicrobial activity was evaluated by the colony counting method adapted from Trandafilović et al. [21]. A membrane disc (5 cm diameter), previously sterilized by exposure to UV light (15 min on each side), was aseptically placed in a tube containing 10 mL of microorganism (*P. aeruginosa* and *S. aureus*) suspension in Mueller–Hinton broth (10^6^ CFU/mL). The suspension of cells in the presence of the membrane was incubated under stirring (150 rpm) at 37 °C for 24 h. Cell viability was recorded immediately before incubation and after 24 h by counting the bacterial colonies formed in Petri dishes containing Müeller–Hinton Agar (Difco, Tucker, GA, USA). The microbial suspensions without any membrane specimens were used as negative controls.

Antimicrobial activity was also evaluated using the agar diffusion assay. In this method, 0.1 mL of suspensions containing 10^6^ CFU/mL of *P. aeruginosa* or *S. aureus* was inoculated into solid Müeller–Hinton culture media (Difco). Membrane samples were aseptically placed on the surface of a Petri dish (9 cm in diameter) containing the inoculated medium, and the set was incubated at 35 °C for 24 h. After this period, the inhibition zone was determined.

#### 2.3.11. Statistical Analysis

The quantitative data are expressed as mean values ± the standard deviation. Analysis of variance and the Tukey test were used to determine statistically significant differences (*p* < 0.05) among the data using the Software Statistica version 7 (TIBCO Software Inc., Palo Alto, CA, USA).

## 3. Results and Discussion

The control and nanocomposite membranes with low and high degree of cross-linking were homogeneous, malleable and easily removed from the casts. In addition, no phase separation was visually detected. With the increase in cross-linking degree, both control and nanocomposite membranes became easier to handle and less soluble in water. The manipulation of nanocomposite membranes was very similar to that of the control membranes.

### 3.1. Nanocomposite Microstructure

**Figure 1:**
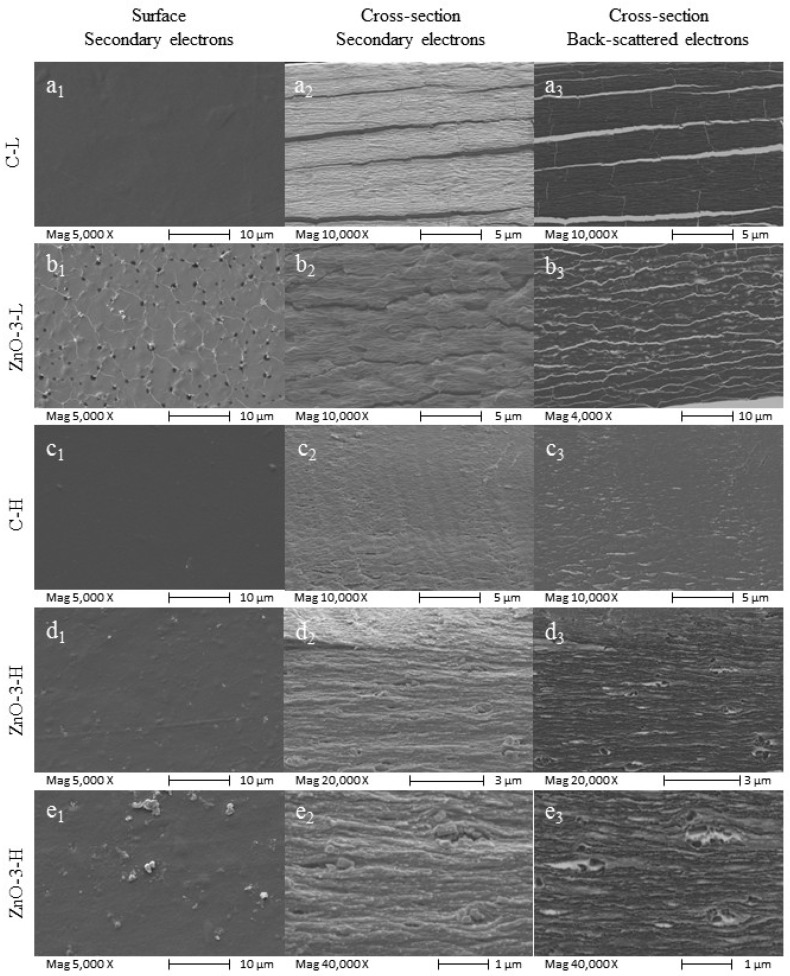
SEM micrographs of control and nanocomposite membranes: (**a**) C-L, (**b**) ZnO-3-L, (**c**) C-H, (**d**) ZnO-3-H and (**e**) ZnO-3-H under higher magnification. In all images: (Subscript 1) surface images obtained with secondary electrons beam; (Subscript 2) cross-section images obtained with secondary electrons beam; (Subscript 3) cross-section images obtained using backscattered electrons beam.

The microstructure of both control and nanocomposite membranes was examined by SEM. Membrane surface and cross-section images, both obtained with secondary electrons beam, and cross-section image obtained with back-scattered electron beam are presented in Figure 1, from left to right, respectively. The visual aspect of the membranes is depicted in Figure S1, as Supplementary Materials.

As seen in Figure 1(a2), the C-L membrane presented an orderly structure typical of the deposition of organized alginate layers. This layered deposition is not observed in the alginate membrane prepared without calcium (images on Supplementary Material, Figure S2), indicating that this organization is due to the calcium cross-linking process. Likewise, the additional increase in cross-linking degree significantly affected the membrane microstructure, smoothing the surface (Figure 1(c1)) and further compacting the polymer matrix, as illustrated in the C-H morphology (Figure 1(c2,c3)). Phase separation may also have occurred in the C-L membrane, which would explain the alginate layer organization.

Backscattered electrons are those emitted from the sample surface due to elastic scattering. Although this electron beam provides a poorer image in terms of resolution, its intensity signal is strongly correlated to the atomic number of the specimen. Thus, the presence of ZnO_nano_ in the cross-section of L and H-membranes is confirmed by the backscattered electrons (Figure 1(b3,d3,e3)). The addition of ZnO_nano_ affected the nanocomposite membrane morphology, with an apparent increase in surface roughness, as evidenced in Figure 1(b1). In the L-membranes, the ZnO_nano_ influenced the membrane microstructure, reducing the degree of organization of the polymer matrix (Figure 1(b2)).

The microstructure of the nanocomposite H-membrane differed from that of the C-H membrane, and it is possible to observe particle agglomeration, as depicted in Figure 1(d1–d3,e1–e3). In Figure 1(e2,e3), obtained at higher magnification, it is possible to observe the relatively large size and heterogeneity of the agglomerates, despite the fairly narrow size distribution of the ZnO_nano_ used in the study, which showed a mean diameter between 83.3 and 272.4 nm (Supplementary Materials Figures S3 and S4).

**Figure 2:**
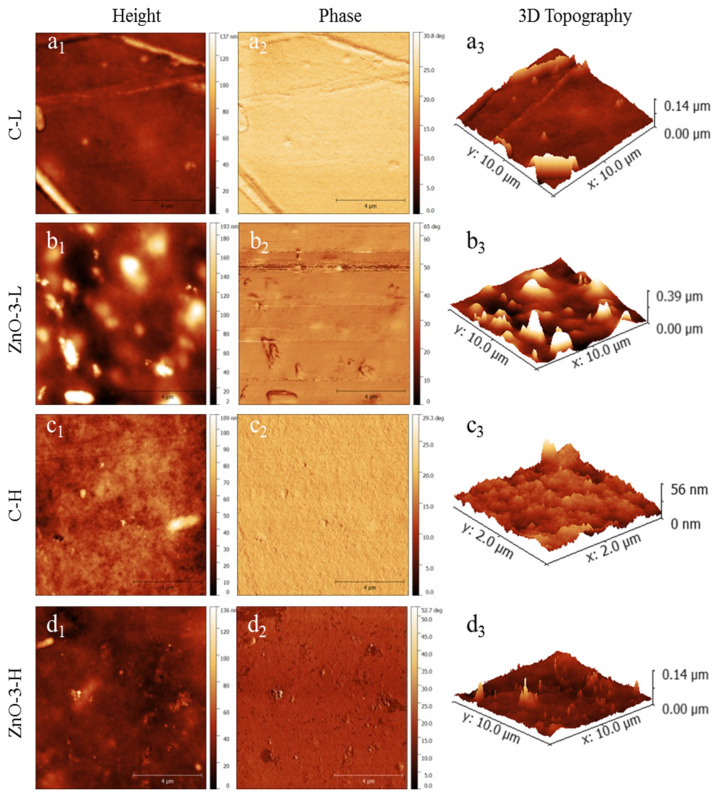
AFM images of control and nanocomposite membranes: (**a**) C-L, (**b**) ZnO-3-L, (**c**) C-H and (**d**) ZnO-3-H. In the images: (Subscript 1) refers to height; (Subscript 2) refers to phase; and (Subscript 3) refers to 3D topography images.

Figure 2 presents the topography of the membranes examined by atomic force microscopy (height, phase and 3D topography). The ZnO-3-L membrane (Figure 2(b1,b3)) presented a rougher surface than the C-L membrane (Figure 2(a1,a3)), which is in accordance with SEM observations. Differences in topography between control and nanocomposite membranes can be attributed to the presence of nanoparticles, as evidenced by the phase images (Figure 2(b2) for ZnO-3L membranes and Figure 2(d2) for ZnO-3-H membrane). The variations in surface topography follows the changes in tip phase, indicating that the tip is in contact with a stiffer material in the same position where the surface height varies [22,23].

The increase on surface roughness of biopolymer nanocomposite membranes has already been reported in other studies involving ZnO-based nanocomposite membranes [24,25]. On the other hand, comparing Figure 1(a1–c1) as well as Figure 1(b1–d1), it is perceptible that the increase in cross-linking degree resulted in a smoother surface, which is also in accordance with the SEM images (Figure 1(c1,d1)). Considering the application as wound dressing, an increase in surface roughness may enhance the adhesion of the membranes to the cells and tissue, thereby stimulating cell proliferation and promoting wound healing [26,27].

### 3.2. X-Ray Diffraction Analysis

**Figure 3:**
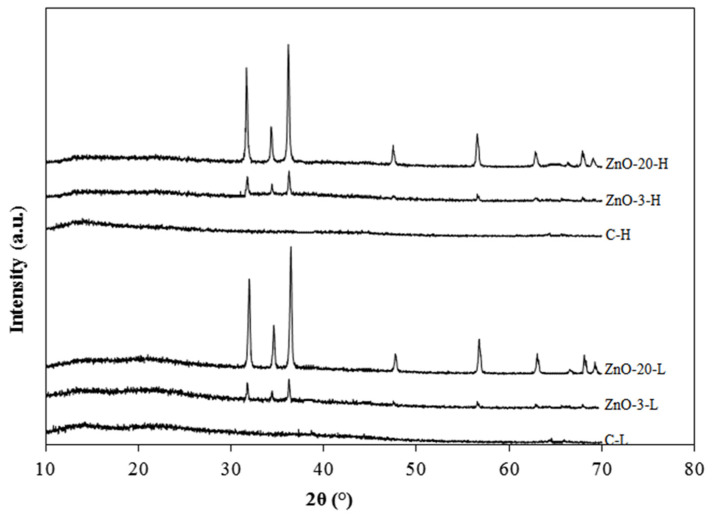
XRD diffraction patterns of control and nanocomposite membranes with low and high degree of cross-linking.

The XRD diffraction patterns of the control and nanocomposite alginate membranes with low and high degree of cross-linking are depicted Figure 3. The two control membranes produced smooth lines without sharp diffraction peaks, confirming their amorphous nature [28]. The membranes containing ZnO_nano_ presented diffraction peaks that increased with the amount of ZnO. These nanocomposite membranes exhibited characteristic diffraction peaks at 2θ equal to 31.6, 34.6, 36.5, 47.7, 56.7, 62.9, 67.9 and 69.2°, corresponding to (100), (002), (101), (102), (110), (103), (200) and (112) planes of zinc oxide, respectively [29,30]. This indicates that the incorporation of ZnO nanoparticles in the alginate matrix did not change their hexagonal wurtzite structure. Furthermore, the peak intensity also increased with increasing ZnO concentration.

### 3.3. FTIR Spectroscopy

**Figure 4:**
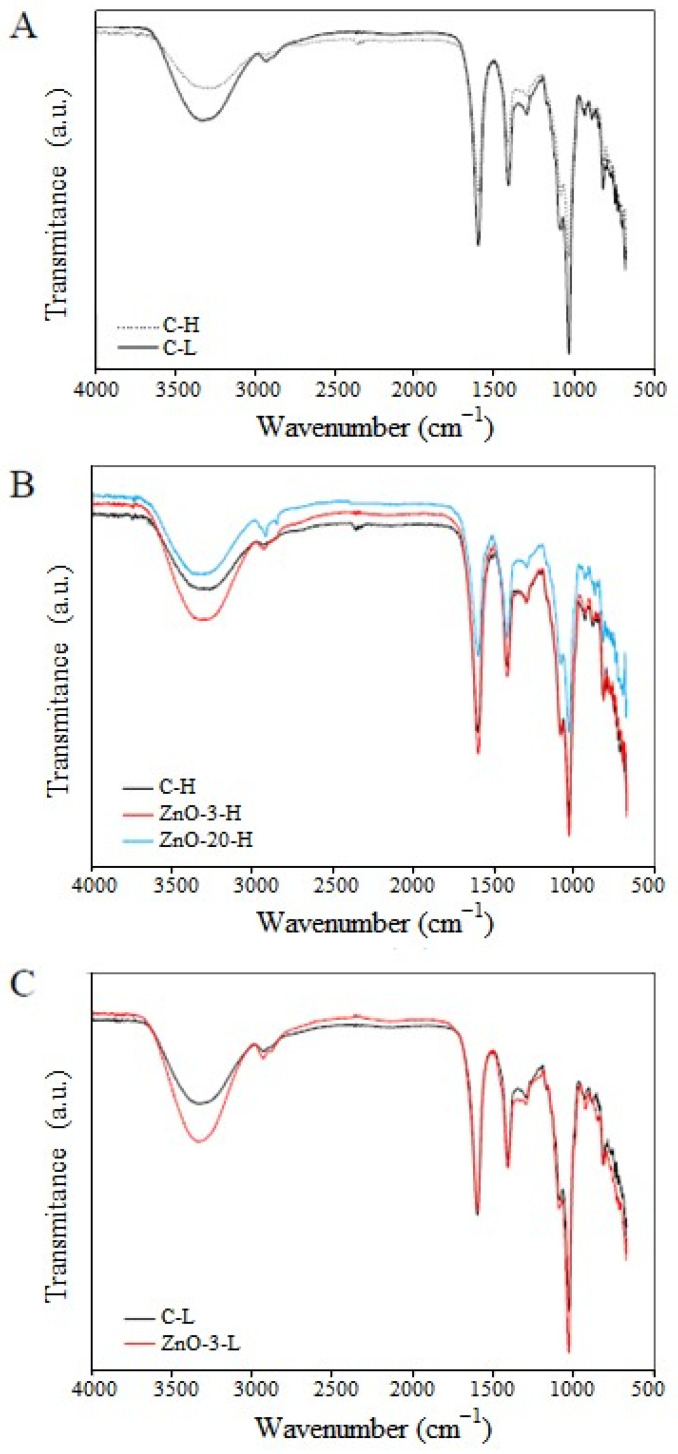
Fourier transform infra-red spectra of control and nanocomposite membranes: (**A**) control membranes with low and high degree of cross-linking, (**B**) nanocomposite membranes with high degree of cross-linking and (**C**) nanocomposite membrane with low degree of cross-linking.

FTIR spectroscopy analysis was carried out to assess specific functional groups and to provide evidence regarding the interaction between the alginate and the nanoparticles. The FTIR spectra of control and nanocomposite membranes containing different concentrations of ZnO_nano_ and different degrees of cross-linking are presented in Figure 4.

There is a broad band of absorption around 3290 and 3346 cm^−1^ which can be attributed to the stretching vibration of hydroxyl (O-H) groups [31]. Stretching of C-H groups was detected at 2930 and 2882 cm^−1^ in the spectra. At 1033 cm^−1^, stretching vibration of C-O groups was also noticed [32,33]. The spectra of C-L membranes also exhibited bands corresponding of the characteristic asymmetrical and symmetrical vibration of carboxyl groups at 1607 cm^−1^ and 1408 cm^−1^, respectively [30]. Spectra of C-H membranes exhibited a shift in asymmetric band to a lower wavenumber (from 1604 cm^−1^ to 1597 cm^−1^) and in the symmetric band to a higher wavenumber (from 1408 cm^−1^ to 1415 cm^−1^). These shifts are indicative of the occurrence of ionic crosslinking [34,35]. In addition, by comparing the absorption spectra of membranes C-L and C-H (Figure 4A), the intensity of the characteristic peaks decreased with increasing cross-linking degree induced by Ca^2+^, due to limitation of chain mobility, which consequently reduced the vibration of functional groups.

In Figure 4B, the absorption spectra of H-membranes containing different contents of ZnO_nano_ are compared. The addition of 3% of ZnO_nano_ to the alginate membrane increased the intensity of the absorption peaks and can be attributed to a possible reduction in the cross-linking density of the alginate chains. However, the increase of ZnO concentration led to a further absorption reduction (as can be seen in the ZnO-20-H absorption spectra), which is most likely due to higher interaction between alginate chains and nanoparticles. A similar trend is observed in the membranes with low degree of cross-linking (Figure 4C).

According to the egg box model, the cross-linking process is a self-cooperative process, similar to a zipper mechanism [36]. In the formation of the junction zones, the coordination of the subsequent calcium ion is facilitated upon the coordination of the previous divalent cation. In the process of nanocomposite membrane fabrication adopted herein, the ZnO nanoparticles are added previously to the cross-linking step with calcium ions. The presence of nanoparticles embedded in the alginate matrix may have hindered the formation of calcium-mediated junction zones, which, on the other hand, are responsible for reducing segmental chain mobility. Therefore, the presence of ZnO_nano_ in the concentration of 3% may have reduced the cross-linking density of the alginate network. However, if the nanoparticles interacted with the polymer chains, an increase in nanoparticle concentration would result in a reduction in vibration intensities. This interaction was evidenced by comparing the absorption spectra of the C-H and ZnO-20-H membranes.

### 3.4. Thermogravimetric Analysis

**Figure 5:**
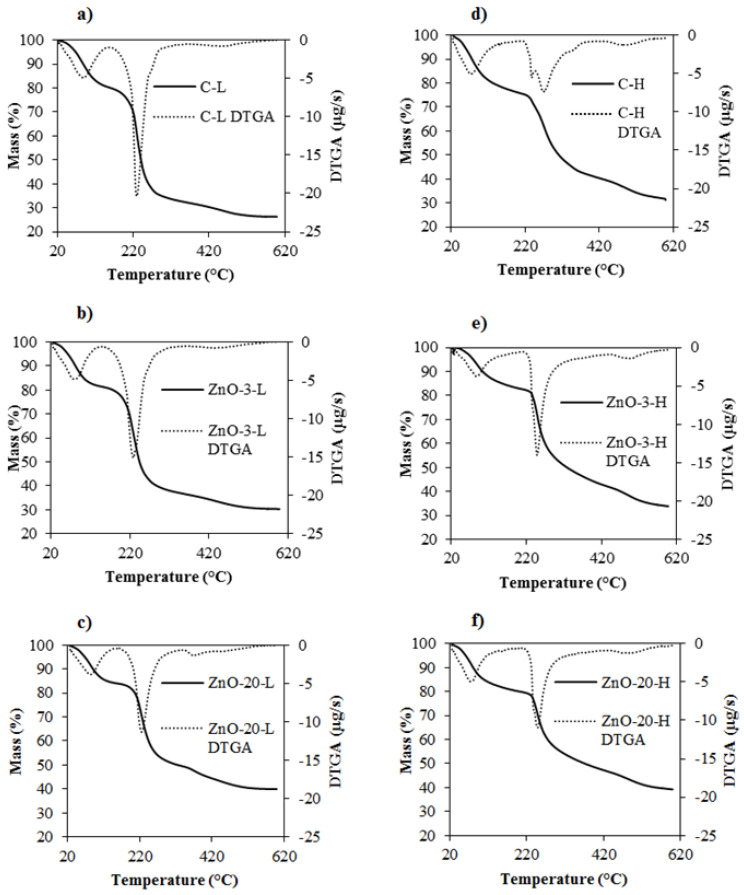
TGA output and derivative form of the TGA curves (DTGA) of control and nanocomposite membranes: (**a**) C-L, (**b**) ZnO-3-L, (**c**) ZnO-20-L, (**d**) C-H, (**e**) ZnO-3H and (**f**) ZnO-20-H.

TGA was performed to evaluate the thermal stability of the control and nanocomposite membranes. The resulting thermograms are presented in Figure 5. Two main thermal events can be observed in membranes both with low and high degrees of cross-linking. The first one, associated with loss of water, occurred between 77 and 89 °C for control and nanocomposite L-membranes and between 73 and 85 °C for the H-membranes [31,32]. The second event showed a peak at 229 °C in the C-L membrane and is related to the decomposition of glycerol and alginate [31]. The temperature of maximum decomposition rate of alginate in C-H membranes, conversely, shifted to 270 °C. This increase in thermal stability can be attributed to the higher degree of cross-linking and corroborates the FTIR results. Giz et al. [31] and dos Santos Araújo [37] also observed that cross-linked alginate chains show higher thermal stability.

The presence of ZnO_nano_ in the L-membranes did not alter the decomposition peak of alginate. However, the addition of ZnO_nano_ to H-membranes decreased the alginate decomposition peak, which was credited to the reduction in the cross-linking density. Vicentini et al. [38] reported increased thermal stability after the addition of ZnO to chitosan-poly(vinyl) membranes, attributing it to a reduction in interatomic distance, thus requiring more energy to decompose the membranes. On the other hand, Shalumon et al. [39] observed that the addition of ZnO nanoparticles to sodium alginate-PVA nanofibers did not contribute to strengthening the thermal stability of the composite.

Therefore, the thermal stability of the resulting composite material will depend on the structure and composition of the polymeric matrix as well as on the interactions between the nanoparticles and the matrix. In the case of alginate-ZnO nanocomposite membranes, the ZnO nanoparticles improve the thermal stability if the degree of cross-linking is low. However, the ZnO presence in the alginate matrix may slow down the cross-linking process if a higher concentration of calcium is used, thus slightly weakening the thermal stability. This reduction in cross-linking density is in agreement with the FTIR spectra observations, since the addition of 3% of ZnO in the H-membrane increased the intensity of absorption peaks.

### 3.5. Mechanical Properties

The values of thickness, tensile strength (TS), elongation at break (E) and Young modulus (YM) of the control and nanocomposite membranes are shown in Table 2.

The decrease in thickness with the increase of calcium concentration is in accordance with SEM observations and is due to the development of a more compact polymeric matrix resulting from more intense crosslinking. Similar trends were observed by Liling et al. [40] for alginate cross-linked with different Ca^+2^ concentrations.

The addition of ZnO_nano_ to alginate membranes did not alter their thicknesses or their mechanical properties. However, the cross-linking with calcium ions markedly affected the mechanical properties, especially the elongation at break and Young’s modulus, due to the development of a more rigid polymeric structure [41]. Specifically for alginate membranes, the increase in the cross-linking degree also leads to a decrease in chain flexibility and mobility, resulting in reduced value of elongation [34,42]. Based on the results presented in Table 2, it is reasonable to infer that the effect of cross-linking on mechanical properties prevails over the effect of ZnO_nano_ addition. In addition, nanoparticle aggregation, as observed in SEM micrographies, reduces the interfacial area between the dispersed and continuum phases in the composite, not improving as expected the mechanical properties of the alginate membranes.

Vicentini et al. [38] developed a nanocomposite membrane of chitosan/PVA with ZnO_nano_ and observed a decrease in TS, which was attributed to a reduction in the interactomic distances between chitosan chains. In alginate membranes, Aristizabal-Gil et al. [43] observed an increase in TS and E with the addition of ZnO and ZnO/CaO nanoparticles up to a certain concentration, beyond which a decrease was noted due to the formation of aggregates.

The mechanical features of wound dressings must ensure their integrity during storage, handling, application and, mostly, use. The skin tensile strength is in the range of 2.5–16 MPa, and its Young’s modulus varies from 6 to 40 GPa [44]. Thus, the developed alginate-ZnO nanocomposite membranes present adequate mechanical properties for application as wound dressings. However, since the H-membranes presented low elongation, their use would not be suitable for articulate regions of the body.

### 3.6. Water Vapor Permeability and Swelling Degree

As indicated in Table 3, water vapor permeability (WPV) results followed a similar trend as the one observed for the mechanical properties: The effect of cross-linking on WVP was far more significant than the increase in the ZnO_nano_ concentration. A decrease in WVP with the increase of the cross-linking degree of alginate with calcium can be attributed to a reduction on chain segmental mobility, which may decrease water permeation through the membrane [40,45].

Motelica et al. [46] obtained alginate nanocomposite membranes with ZnO_nano_ without cross-linking and noticed that the incorporation of ZnO significantly reduced the WVP by increasing the pathways for water molecules. However, Aristizabal-Gil et al. [43] found an increase in WVP after the incorporation of ZnO and ZnO/CaO nanoparticles in alginate membranes, attributing it to the disruption of the polymeric matrix by agglomerates, which created an easier pathway for water vapor migration.

An ideal dressing should control the evaporative water loss at an optimal rate to avoid excessive dehydration or fluid accumulation. The water vapor transmission rate (WVTR) of skin is approximately 0.003 gm^−2^s^−1^ for first degree burns and goes up to 0.06 gm^−2^s^−1^ for granulating wounds [47,48], while normal skin has a WVTR of about 0.002 gm^−2^s^−^1 [49]. The average WVTR found for L-membranes and H-membranes were 0.002 and 0.001 gm^−2^s^−^1, respectively, so they could be recommended for wounds with low WVR demand or low to medium exudating wounds.

Membranes with a low degree of cross-linking (L-membranes) showed significantly higher moisture content (MC) than membranes subjected to the second stage of cross-linking (H-membranes). This behavior suggests that a lower degree of cross-linking allows more charged sites to be accessible to water molecules, consistent with WVP results. The incorporation of ZnO_nano_ did not change the moisture content of membranes of the same group (L or H-membranes).

The swelling studies showed that the control and nanocomposite membranes behave differently depending on the fluid composition. Swelling degrees of L-membranes are not reported because this membrane formulation is completely solubilized in all the three tested fluids. The swelling of the three types of H-membranes was similar when in contact with each one of the fluids, but the swelling in FBS was 7 times larger than in water, probably due to the pH difference and possible displacement of Ca^+2^. When immersed in PBS, the swelling of nanocomposite membranes was several orders of magnitude larger than in water and the control H-membrane was fully solubilized. This occurred possibly due to calcium displacement from the reticulated matrix by phosphate groups, resulting in the collapsing of the C-H membrane. The swelled nanocomposite membranes, however, were stable and maintained the integrity during handling.

These results show a promising performance of alginate-based nanocomposite materials for applications such as wound dressings. When in contact with fluids containing sodium, such as wound exudates, alginate-ZnO nanocomposite membranes can keep their integrity while offering a high level of absorption, ideal for medium to medium-high exudating wounds.

### 3.7. In Vitro Antimicrobial Activity

**Figure 6:**
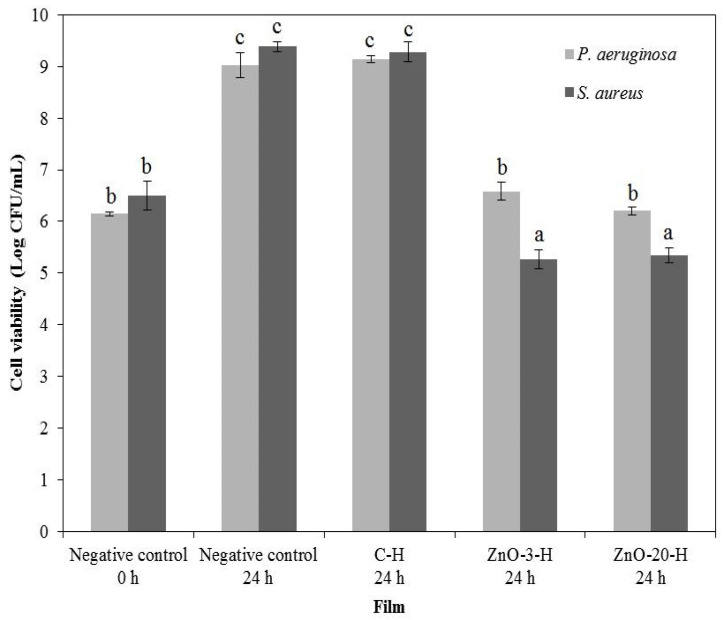
Antimicrobial activity of control and nanocomposite H-membranes against *P. aeruginosa* and *S. aureus*. Bars marked with the same letter indicate no significant differences (*p* < 0.05) as determined by Tukey’s test.

As the L-membranes collapsed in aqueous media, which would enhance the ZnO_nano_ availability to inhibit microbial growth, the in vitro antimicrobial activity assessment of nanocomposite membranes was performed in a more conservative approach, using only the H-membranes, and the results are illustrated in Figure 6.

The control membrane presented no activity against both bacteria strains. The nanocomposite membranes reduced the total bacteria cell counting of *S. aureus*, and a bacteriostatic effect against *P. aeruginosa* was detected. ZnO_nano_ barely diffused out of the nanocomposite membrane, as observed through the agar diffusion assay, and growth inhibition occurred by contact (data in Supplementary Materials, Figures S5–S8).

Pasquet et al. [50] also observed that ZnO_nano_ hardly diffuses out of cellulose membrane discs. The absence of ZnO_nano_ diffusion may be related to a number of factors, such as nanoparticle agglomeration, particle affinity towards the alginate matrix and the relatively packed nature of the membrane, which potentially contribute to hinder nanoparticle transport to the culture media.

ZnO antibacterial activity is most likely due to the formation of reactive species of oxygen (ROS), such as H_2_O_2_, O_2_−, OH∙ and OH−, that may damage bacterial cell walls and increase the oxidative stress, resulting in negative effects in proteins, lipids and DNA [51].

*S. aureus* was more susceptible to ZnO_nano_ than *P. aeruginosa*, which is in agreement with the works of Pasquet et al. [50] and Ann et al. [51]. Pasquet et al. [50] found that the minimum inhibitory concentration (MIC) of ZnO_nano_ for *P. aeruginosa* was five times greater than the MIC for *S. aureus*, which was attributed to the presence of cytochrome oxidase in *P. aeruginosa* that quickly reduces ROS such as H_2_O_2_ to water. *S. aureus* also expresses catalase, which directs the reduction in hydrogen peroxide to diminish the oxidative stress, but in limited quantities not sufficient to withstand the oxidative stress imposed by ZnO_nano_ [52]. Other authors also detected antimicrobial activity of ZnO_nano_ against *S. aureus* [13,38].

The alginate-ZnO nanocomposite membranes were then capable of controlling the bacterial population, which gives support to their use as wound dressings to prevent microbial invasion and proliferation.

### 3.8. Color Properties and Opacity

The results of the color parameters and the opacity of the membranes are summarized in Table 4, and the visual aspect of the H-membranes is shown in Figure S1 of the Supplementary Material.

The color attributes and the opacity were significantly affected by the content of ZnO_nano_ in the nanocomposite membranes. The control membranes were transparent, while the membranes with ZnO nanoparticles presented a whitish or yellowish appearance. Both Hue and Chroma were similarly affected by the increase in ZnO_nano_ concentration, independently of the cross-linking degree. The increment in ZnO_nano_ from 3 to 20% shifted the tonality (Hue) from blue to yellow and increased the tonality intensity (Chroma). Comparing the nanocomposite membranes (low and high cross-linking) to their respective control membranes, the color difference (ΔE*) gradually increased with the addition of higher amounts of ZnO_nano_. The opacity of the membranes was affected by the ZnO_nano_ concentration and cross-linking degree. The increase of calcium content decreased the opacity of C-L and C-H membranes, a trend that was expected due to the formation of a more compact polymeric matrix, reducing the light diffraction and refraction. The decrease in transparency of the nanocomposite membranes can also be ascribed to the reduced distance between nanoparticles due to polymer packing.

Espitia et al. [17] also observed that the increase in ZnO_nano_ concentration in CMC-ZnO nanocomposite membranes leads to an increase in opacity. In addition, increasing ZnO concentration in these membranes was responsible for reducing the values of both parameters L* and b*, altering the luminosity and the color (blue/green) of the membranes. Aristizabal-Gil et al. [43] observed a decrease in membrane opacity with increasing ZnO nanoparticle concentration in alginate membranes, which was attributed to the light-scattering effect of the nanoparticles, reducing light transmission through the polymeric matrix.

For skin lesions, antimicrobial activity and biocompatibility play a key role when choosing the material for a specific treatment. However, when considering membranes with comparable antimicrobial activity, like the case of the membranes containing 3 and 20% of the ZnO_nano_, data regarding the wound dressing visual properties could also influence the decision of field professionals. In this case, the membrane ZnO-3-H could aid in monitoring of the healing process, due to its highest transparency, without reduction in the antimicrobial effect.

## 4. Conclusions

Alginate-ZnO nanocomposite membranes were prepared at different cross-linking degrees and with different nanoparticle content. Nanoparticle incorporation itself may have changed the cross-linking density, as observed by FTIR and TGA analysis. The cross-linking degree was determinant to membrane properties, such as water vapor permeability, mechanical properties and thermal stability, while color properties and opacity were more affected by the ZnO_nano_ content. Alginate-ZnO nanocomposite membranes inhibited the proliferation of *P. aeruginosa* and *S. aureus*, and this feature associated with the physicochemical and mechanical properties of the membranes as well as their improved liquid uptake ability turn this material suitable for application as wound dressings. The formulation containing 3% of ZnO_nano_ is recommended, as this concentration provided sound antimicrobial activity, and no significant improvement in dressing functional characteristics was found when 20% of ZnO_nano_ was added to the membrane.

## Figures and Tables

**Table 1:** Membrane formulations and denominations.

Membrane Formulation *	CaCl_2_⋅2∙H_2_O Concentration (%)		ZnO_nano_/Sodium Alginate (m/m)
	1st Stage	2nd Stage	
C-L	0.8	-	0.00
ZnO-3-L	0.8	-	0.03
ZnO-20-L	0.8	-	0.20
C-H	0.8	3.0%	0.00
ZnO-3-H	0.8	3.0%	0.03
ZnO-20-H	0.8	3.0%	0.20

* C: control membranes; L: low cross-linking degree; H: high cross-linking degree.

**Table 2:** Thickness, tensile strength (TS), elongation at break (E) and Young’s modulus (Y) of control and nanocomposite membranes.

Membrane Formulation	Thickness (µm)	TS (MPa)	E (%)	YM (MPa)
C-L	43.8 ± 4.2 ^b^	15.8 ± 2.2 ^a^	27.2 ± 8.1 ^b^	175 ± 38 ^a^
ZnO-3-L	46.5 ± 3.5 ^b^	15.1 ± 2.2 ^a^	27.2 ± 8.1 ^b^	175 ± 38 ^a^
ZnO-20-L	43.0 ± 4.6 ^b^	17.2 ± 3.1 ^a^	23.0 ± 8.4 ^b^	168 ± 33 ^a^
C-H	30.5 ± 4.1 ^a^	62.4 ± 14.0 ^b^	1.7 ± 0.5 ^a^	5001 ± 808 ^b^
ZnO-3-H	33.1 ± 3.5 ^a^	61.7 ± 9.8 ^b^	1.5 ± 0.4 ^a^	4797 ± 688 ^b^
ZnO-20-H	36.1 ± 3.9 ^a^	62.8 ± 10.6 ^b^	2.3 ± 0.8 ^a^	4313 ± 531 ^b^

The same superscript letter within the same column indicates no significant differences (*p* < 0.05) as determined by Tukey’s test.

**Table 3:** Water vapor permeability (WVP), moisture content (MC) and swelling degree (SD in water, PBS and FBS) of control and nanocomposite membranes.

Membrane Formulation	WVP (×10^−14^ gm^−1^s^−1^Pa^−1^)	MC (%)	SD H_2_O (%)	SD PBS (%)	SD FBS (%)
C-L	9.66 ± 1.46 ^b^	33.8 ± 2.7 ^b^	CS	CS	CS
ZnO-3-L	10.82 ± 0.30 ^b^	35.3 ± 1.2 ^b^	CS	CS	CS
ZnO-20-L	12.32 ± 0.98 ^b^	32.5 ± 0.5 ^b^	CS	CS	CS
C-H	3.65 ± 0.43 ^a^	23.2 ± 2.0 ^a^	132.7 ± 7.1 ^a^	CS	905.6 ± 49.7 ^a^
ZnO-3-H	3.83 ± 0.42 ^a^	20.4 ± 0.7 ^a^	130.4 ± 12.9 ^a^	2786.3 ± 461.7 ^a^	971.5 ± 51.2 ^a^
ZnO-20-H	3.76 ± 0.06 ^a^	19.3 ± 2.1 ^a^	120.0 ± 6.0 ^a^	3107.3 ± 332.5 ^b^	957.9 ± 92.3 ^a^

The same superscript letter within the same column indicates no significant differences (*p* < 0.05) as determined by Tukey’s test. CS: Complete solubilization.

**Table 4:** Color properties and opacity (OP) of control and nanocomposite membranes.

Membrane Formulation	Hue	Chroma	ΔE*	OP (%)
C-L	−84.07 ± 0.72 ^a^	1.69 ± 0.37 ^a^	-	3.80 ± 0.12 ^b^
ZnO-3-L	−86.26 ± 0.05 ^b^	6.70 ± 0.55 ^b^	5.27	6.20 ± 0.25 ^c^
ZnO-20-L	86.59 ± 0.20 ^e^	15.21 ± 1.80 ^c^	18.81	17.77 ± 0.14 ^e^
C-H	−88.14 ± 0.46 ^c^	1.59 ± 0.23 ^a^	-	1.37 ± 0.47 ^a^
ZnO-3-H	−88.64 ± 0.37 ^c^	6.64 ± 0.74 ^b^	5.82	10.07 ± 0.64 ^d^
ZnO-20-H	84.16 ± 0.69 ^d^	15.14 ± 1.92 ^c^	19.33	22.1 ± 0.15 ^f^

The same superscript letter within the same column indicates no significant differences (*p* < 0.05) as determined by Tukey’s test.

## Data Availability

The original contributions presented in the study are included in the article. Further inquiries can be directed to the corresponding author.

## Supplementary Materials

The following supporting information can be downloaded at: https://www.mdpi.com/article/10.3390/membranes15040108/s1, Figure S1: Visual aspect of the control and nanocomposite membranes: (a) C-H, (b) ZnO-3-H, (c) ZnO-20-H; Figure S2: Cross-section of alginate membrane prepared with glycerol and without calcium cross-linking at different magnifications; Figure S3. Estimation of the diameter of ZnO_nano_ particles inside the alginate matrix (micrographs from secondary electron beam) at different magnifications; Figure S4: ZnO_nano_ diameter estimation inside the alginate matrix (micrograph from backscattered electron beam); Figure S5: Agar diffusion assay: Antimicrobial activity against *Pseudomonas aeruginosa* of C-H membrane; Figure S6: Agar diffusion assay: Antimicrobial activity against *Pseudomonas aeruginosa* of ZnO-3-H membrane; Figure S7: Agar diffusion assay: Antimicrobial activity against *Staphylococcus aureus* of C-H membrane; Figure S8: Agar diffusion assay: Antimicrobial activity against *Staphylococcus aureus* of ZnO-3-H membrane.
