# Supplementary material for: Influence of Nanoparticle Content and Cross-Linking Degree on Functional Attributes of Calcium Alginate-ZnO Nanocomposite Wound Dressings
Source: Membranes (Basel). 2025 Apr 1;15(4):108. doi: 10.3390/membranes15040108 (PMC12029479; doi:10.3390/membranes15040108)
Supplement: Supplementary file 1 [file membranes-15-00108-s001.zip › membranes-3390823-supplementary.pdf]

Supplementary Materials

Influence of Nanoparticle Content and Cross-Linking Degree on Functional Attributes of Calcium Alginate-ZnO Nanocomposite Wound Dressings

Sergio Henrique Toledo e Silva [1], Andrea Cristiane Krause Bierhalz [2,*] and Ângela Maria Moraes [1,*]

[1] School of Chemical Engineering, University of Campinas, Avenida Albert Einstein, 500, Cidade Universitária Zeferino Vaz, Campinas 13083-852, São Paulo, Brazil; sergio.toledo@ivv.fraunhofer.de
[2] Department of Textile Engineering, Federal University of Santa Catarina, Rua João Pessoa 2750, Blumenau 89036-004, Santa Catarina, Brazil
* Correspondence: andrea.krause@ufsc.br (A.C.K.B.); ammoraes@unicamp.br (Â.M.M.); Tel.: +55-47-33895177 (A.C.K.B.); +55-19-35213920 (Â.M.M.)

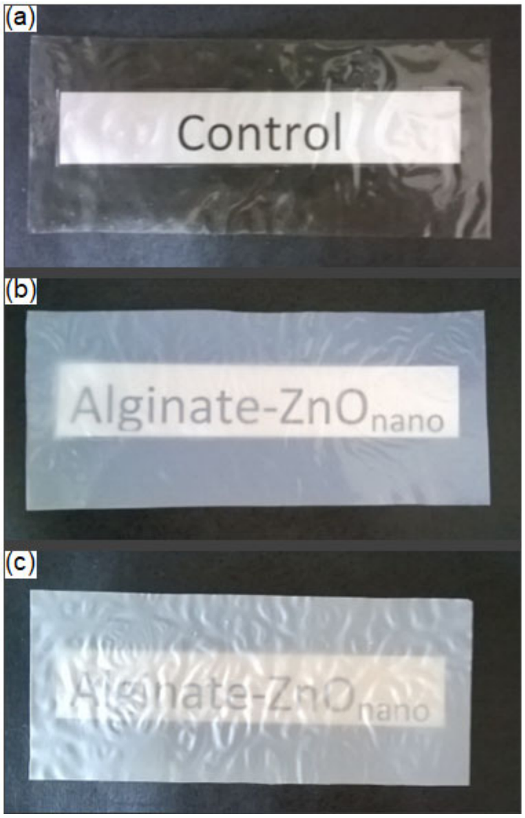

Figure S1. Visual aspect of the control and nanocomposite membranes: (**a**) C-H, (**b**) ZnO-3-H, (**c**) ZnO-20-H.

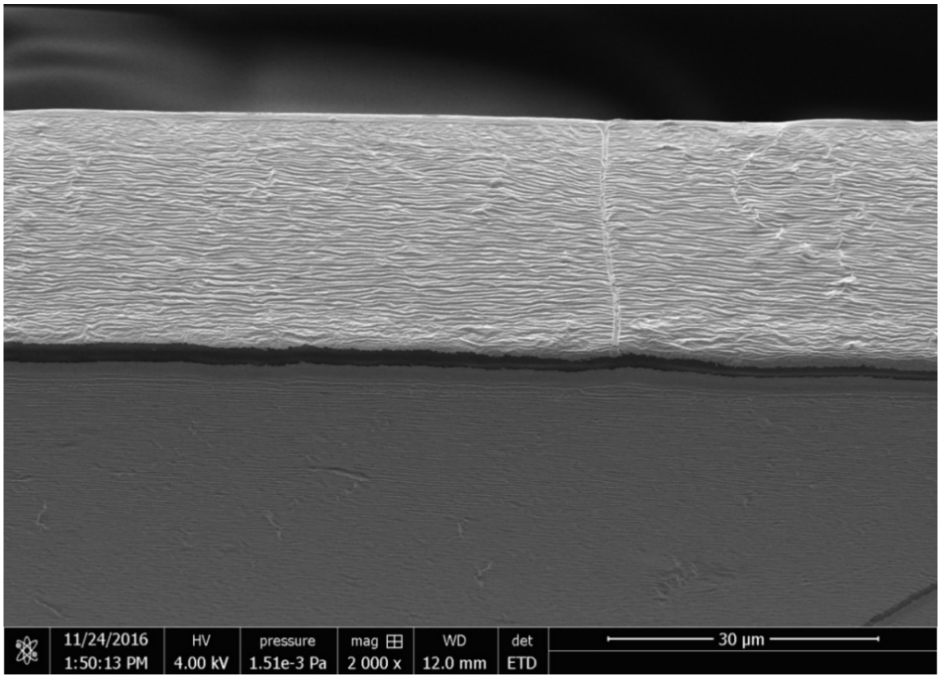

(a)

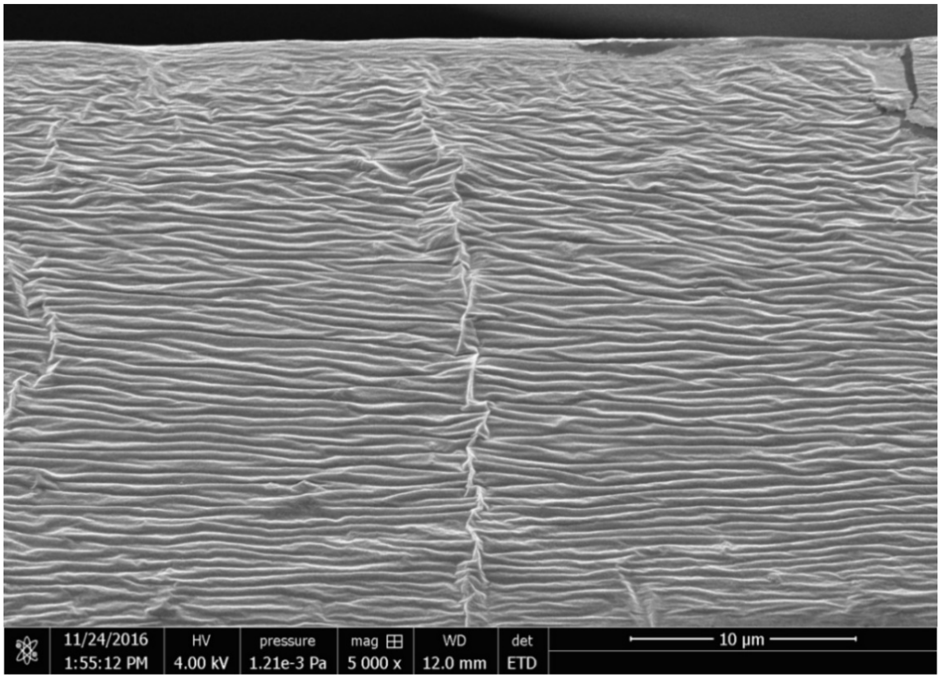

(b)

Figure S2. Cross-section of alginate membrane prepared with glycerol and without calcium cross-linking at different magnifications (**a**) ×2000 and (**b**) ×5000.

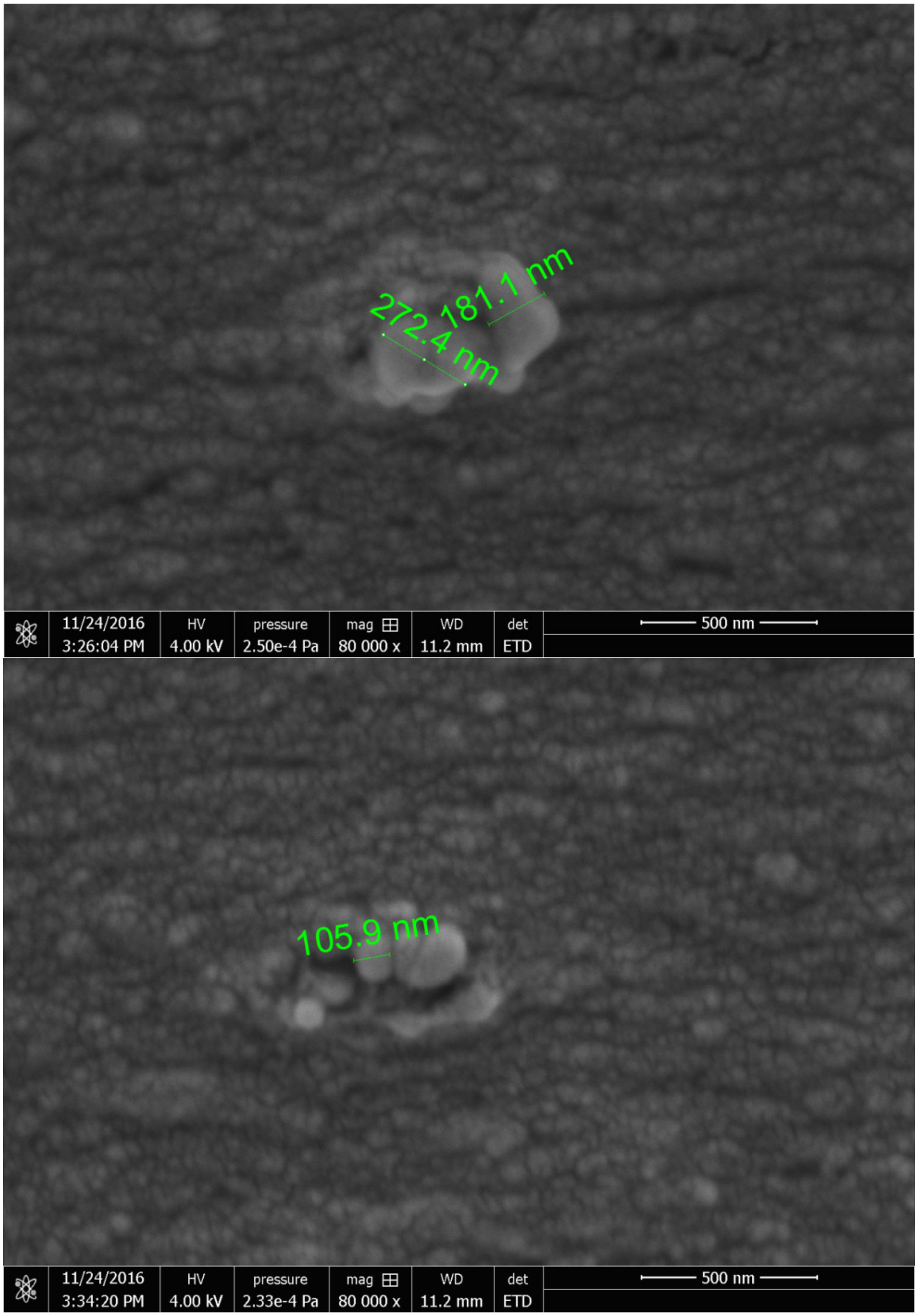

Figure S3. Estimation of the diameter of ZnO_{nano} particles inside the alginate matrix (micrographs from secondary electron beam)

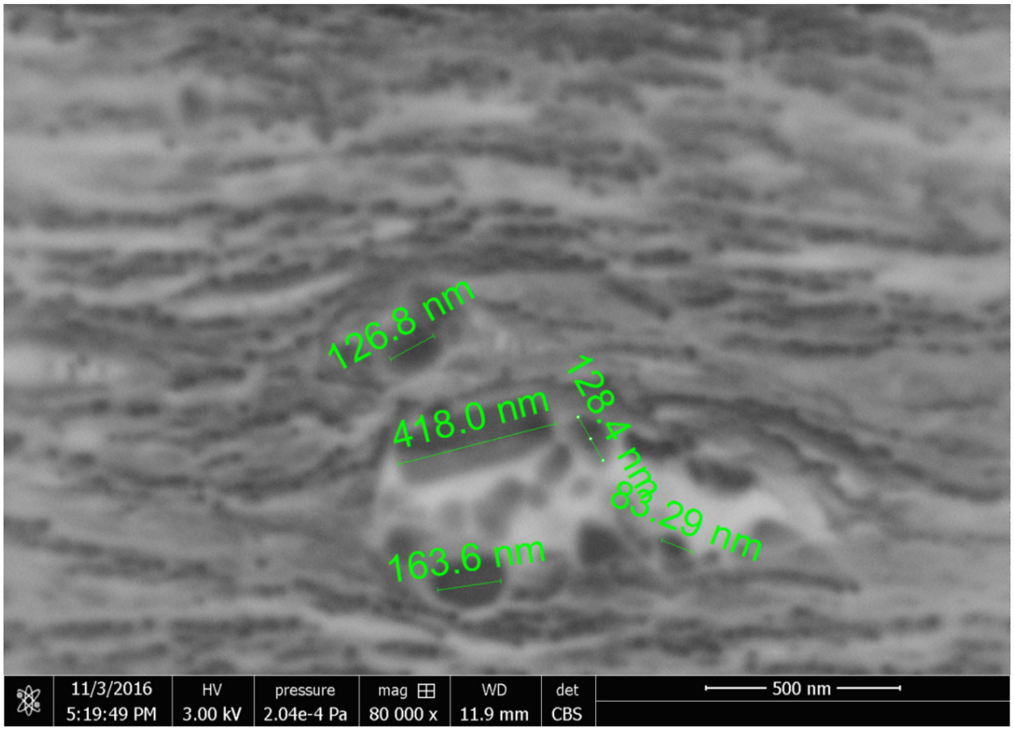

Figure S4. ZnO_{nano} diameter estimation inside the alginate matrix (micrograph from backscattered electron beam).

Agar diffusion assay: Antimicrobial activity against *Pseudomonas aeruginosa* and *Staphylococcus aureus*. Microbial growth is observed under C-H membrane and not under the nanocomposite membrane.

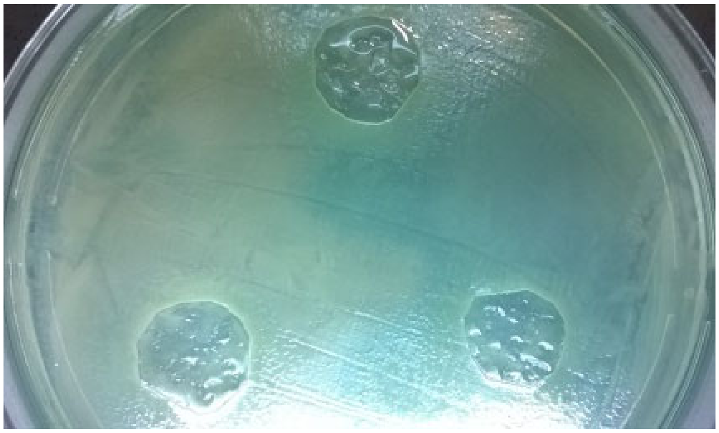

Figure S5. Agar diffusion assay: Antimicrobial activity against *Pseudomonas aeruginosa* of C-H membrane.

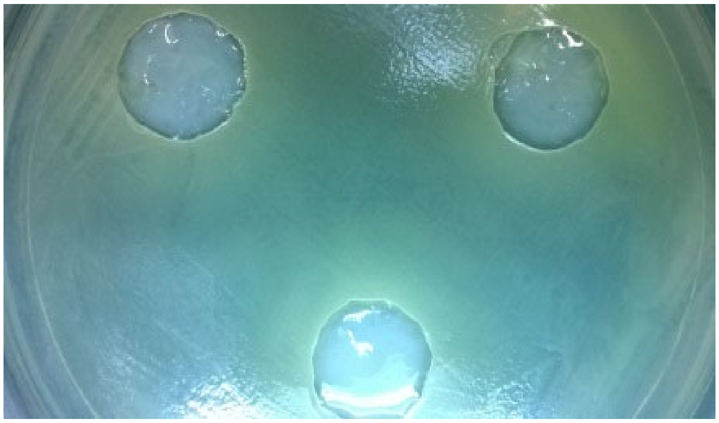

Figure S6. Agar diffusion assay: Antimicrobial activity against *Pseudomonas aeruginosa* of ZnO-3-H membrane.

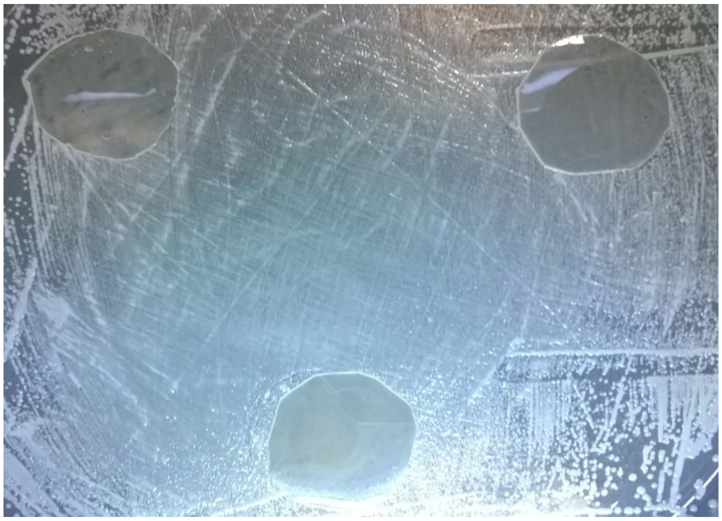

Figure S7. Agar diffusion assay: Antimicrobial activity against *Staphylococcus aureus* of C-H membrane.

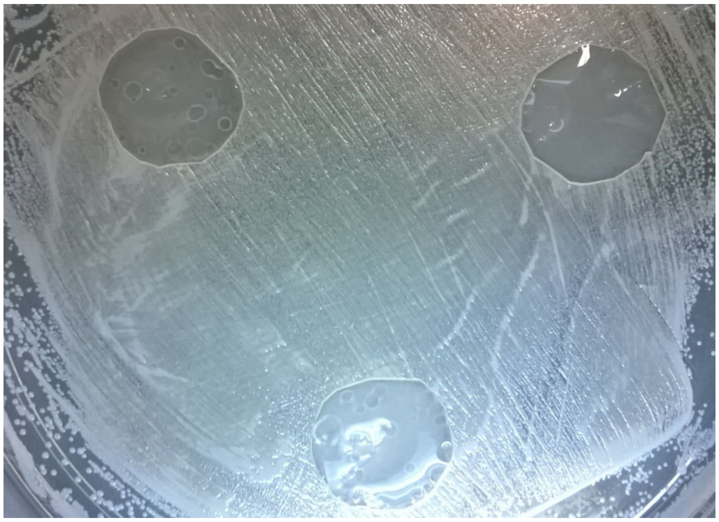

Figure S8. Agar diffusion assay: Antimicrobial activity against *Staphylococcus aureus* of ZnO-3-H membrane.
